# Laser Therapy Versus Conventional Treatments in Oral Leukoplakia: A Systematic Review of Efficacy, Recurrence, and Postoperative Outcomes

**DOI:** 10.1155/ijod/2130179

**Published:** 2026-01-22

**Authors:** Gianna Dipalma, Angelo Michele Inchingolo, Pietro Lauria, Pierluigi Marotti, Silvia Chieppa, Daniela Di Venere, Andrea Palermo, Massimo Corsalini, Francesco Inchingolo, Alessio Danilo Inchingolo

**Affiliations:** ^1^ Department of Interdisciplinary Medicine, University of Bari “Aldo Moro”, Bari, 70121, Italy, uniba.it; ^2^ Department of Experimental Medicine, University of Salento, Lecce, 73100, Italy, unisalento.it

**Keywords:** ablative lasers, fractional lasers, laser-based therapies, lesion regression, oral leukoplakia, oral squamous cell carcinoma, photodynamic therapy (PDT), potentially malignant disorder

## Abstract

Oral leukoplakia (OL) is a potentially malignant disorder characterized by white lesions on the oral mucosa, with a variable risk of progression to oral squamous cell carcinoma (OSCC). Its management remains challenging due to the potential for recurrence and malignant transformation. In recent years, laser‐based therapies have emerged as promising alternatives to traditional treatments such as surgical excision and cryotherapy. This systematic review evaluates the efficacy and safety of various laser technologies, including ablative lasers, fractional lasers, and photodynamic therapy (PDT)‐assisted laser treatments, for the management of OL. The review focuses on key clinical outcomes such as lesion regression rates, recurrence reduction, and the incidence of adverse effects. Evidence suggests that laser therapies not only achieve significant lesion regression but also minimize posttreatment discomfort and preserve healthy tissue structure. Among the laser modalities studied, PDT‐assisted treatments have shown particular promise in reducing recurrence rates over long‐term follow‐up. Furthermore, fractional laser‐assisted PDT has demonstrated high clinical efficacy with excellent tolerability, suggesting its potential as a front‐line treatment option. Despite these encouraging results, further research with larger patient cohorts and longer follow‐up periods is necessary to establish standardized treatment protocols. This review supports the adoption of laser‐based treatments as effective and minimally invasive options for managing OL while reducing the risk of malignant progression.

## 1. Introduction

Oral leukoplakia (OL) is among the most common potentially malignant disorders affecting the oral cavity. It is characterized by white plaques or patches that cannot be scraped off and lack a clear underlying cause [1–3]. The condition is observed in ~1% to 4% of the population, with a highly variable risk of progressing to oral squamous cell carcinoma (OSCC). Although OL presents in different clinical forms, its malignant transformation potential is what makes it clinically significant. Early detection and timely management are crucial in reducing cancer risk, emphasizing the need for effective treatment strategies [4–7].

The development of OL is influenced by multiple factors, including genetic, environmental, and behavioral components [8]. The primary risk factors include prolonged tobacco use in both smoking and smokeless forms, excessive alcohol consumption, and infection with human papillomavirus (HPV), particularly Types 16 and 18 [6, 7, 9, 10]. These well‐established contributors significantly elevate the risk of malignancy [11–13].

Although well‐established risk factors such as tobacco and alcohol use are commonly associated with OL, the disorder can also occur in individuals without these exposures [14, 15]. Epidemiological studies suggest that genetic predispositions or subtle environmental influences may contribute to disease onset in such cases [16, 17]. Additional research indicates that immune system variations and other, less understood biological mechanisms could play a role in the development of OL [18, 19].

For instance, immune system dysfunction, nutritional deficiencies, and chronic inflammatory conditions of the oral mucosa may also play a role. A comprehensive understanding of the disease thus necessitates consideration of both established and potential risk factors [20–23].

Clinically, OL varies in presentation, ranging from small, mild white patches to larger, ulcerated lesions with a heightened risk of malignant transformation [24–27]. Due to this variability, monitoring lesion size, appearance, and progression rate is vital in determining the most appropriate treatment approach [28]. One of the primary challenges in OL management is its unpredictable course. While some lesions remain benign over time, others may evolve into OSCC, highlighting the importance of vigilant observation and timely intervention.

Traditional treatment options for OL include surgical excision, cryotherapy, and pharmacological interventions. Surgical removal is considered the gold standard for treating high‐risk lesions, but it poses risks such as scarring, functional impairment, and a high recurrence rate, especially if the lesion is not completely excised [29–32]. Cryotherapy, which works by freezing and destroying abnormal tissue, also has drawbacks, including pain and potential tissue damage that may affect both function and aesthetics. Pharmacological approaches, such as topical corticosteroids or retinoids, have been explored [28].

Although these treatment modalities are widely used, several analyses have shown that their overall effectiveness can be limited, particularly when lesion characteristics reduce therapeutic responsiveness [33, 34]. Further evidence indicates that many interventions are frequently accompanied by postoperative discomfort or other adverse effects, which may constrain their clinical applicability and long‐term acceptance by patients [35, 36]. Additional reports have also highlighted variable healing patterns and occasional functional disturbances, underscoring the need for careful selection and individualized planning [37, 38].

Despite the availability of current therapeutic options, several analyses have shown that recurrence remains a significant concern, particularly in patients presenting extensive or high‐risk lesions [39, 40]. Additional evaluations have reported that persistent epithelial alterations can undermine long‐term disease stability, contributing to relapse even after apparently successful treatment [41, 42]. Other studies further indicate that lesions with irregular or multifocal patterns show limited therapeutic durability, with a notable tendency to reappear during follow‐up [43, 44]. Overall, evidence suggests that outcomes remain less stable in individuals classified as high‐risk, confirming the need for careful monitoring after treatment [45].

Given these limitations, laser‐based therapies have emerged as a promising alternative, offering several advantages over conventional treatments. Lasers, including carbon dioxide (CO_2_) lasers, diode lasers, and ablative fractional lasers, enable precise lesion removal with minimal damage to surrounding healthy tissue, thereby reducing the risk of complications such as scarring and functional impairment [46–50].

Laser treatment has been consistently associated with reduced postoperative pain, providing patients with a more comfortable recovery experience [51, 52]. Evidence also suggests that laser‐assisted procedures can accelerate healing, promoting faster restoration of normal oral function [53, 54]. Studies indicate that tissue regeneration occurs more efficiently following laser therapy, which may contribute to shorter recovery times and improved clinical outcomes [55, 56]. Patient‐reported outcomes highlight greater satisfaction with laser interventions due to decreased inflammation and postoperative discomfort [57, 58].

Photodynamic therapy (PDT) has recently gained attention as an adjunct to laser treatment, particularly for minimizing OL recurrence. PDT involves applying a photosensitizing agent, such as 5‐aminolevulinic acid (ALA), which is absorbed by abnormal cells and subsequently activated by a specific light wavelength. This selective destruction of affected cells promotes lesion healing while preserving surrounding tissue. When combined with laser activation, PDT has demonstrated effectiveness in reducing lesion size, normalizing epithelial tissue, and decreasing the likelihood of malignant progression.

PDT has been shown to reduce the incidence of adverse effects compared to conventional treatments, enhancing patient comfort [59, 60]. Some studies report that PDT preserves surrounding healthy tissue while effectively targeting leukoplakic lesions [61, 62]. Clinical evidence also indicates that PDT minimizes postoperative inflammation and promotes faster mucosal recovery [63, 64]. Overall, this approach offers patients a treatment option that balances efficacy with functional and aesthetic considerations [65].

With these advancements, there is growing interest in further exploring laser‐based therapies for OL management. This systematic review aims to assess the current evidence on the safety, efficacy, and clinical outcomes of various laser treatments, including CO_2_ lasers, diode lasers, and PDT‐assisted laser therapies [66]. By analyzing existing studies, this review seeks to provide a comprehensive overview of how laser technology can be effectively incorporated into clinical practice, highlighting its benefits, limitations, and future research directions [60].

In addition to evaluating treatment efficacy, this review aims to identify key clinical outcomes associated with laser therapy, such as lesion regression, recurrence reduction, and long‐term follow‐up data [67]. While initial studies indicate promising results, larger and more rigorous clinical trials are needed to establish the definitive role of laser treatments in OL management. Long‐term data are particularly crucial in determining whether these therapies provide sustained benefits and significantly lower the risk of malignant transformation over time [68–71].

Furthermore, as with any medical intervention, cost and accessibility remain important factors [28]. Although laser treatments are generally more expensive than traditional methods, their potential to reduce complications, accelerate recovery, and enhance patient satisfaction may make them a more cost‐effective option in the long term [67, 72–74]. As laser technology continues to evolve, more affordable and widely accessible options are expected to emerge, making these innovative treatments available to a broader patient population [75–79].

In conclusion, OL presents a considerable clinical challenge due to its potential for malignant transformation and the limitations of current treatment options [80, 81]. Laser‐based therapies, especially when combined with PDT, have shown great potential as effective and minimally invasive alternatives [82–85]. This review aims to consolidate the latest research on these technologies to inform clinical practice and guide future investigations into their role in OL management [86]. Through continued research and technological advancements, it is anticipated that more precise, efficient, and accessible treatments will become available, ultimately improving patient outcomes and reducing the risk of cancer progression [87–90].

## 2. Materials and Methods

### 2.1. Search Processing

The current systematic review followed the PRISMA and International Prospective Register of Systematic Review Registry procedures. It was registered with the International Prospective Register of Systematic Review (PROSPERO ID 1010154).

The following databases: PubMed, Web of Science, and Scopus, were examined from January 15, 2025 to January 22, 2025, to search articles of the last 15 years. The search strategy was created by combining terms relevant to the study’s purpose.

The following Boolean keywords were applied: ((Oral Leukoplakia) AND (Laser Therapy) AND (Nd:Yag Laser) AND (CO_2_ Laser)).

### 2.2. Inclusion End Exclusion Criteria

The reviewers worked in a group to assess all relevant studies that analyzed or compared the effects of laser on OL, according to the following inclusion criteria:

• Studies that did the research “in vivo” or in “humans;”
• Case‐controls studies, cohort studies, RCTs;
• Studies that were published in the last 15 years.

Studies that fulfill at least one of the following exclusion criteria were excluded: reviews, case reports, and series, letters to the authors; animal models; in vitro studies.

#### 2.2.1. PICO Question

The PICO question addressed was:

“Among patients diagnosed clinically and histologically with OL, how do laser‐based treatments (ablative, fractional, and laser‐assisted PDT) compare to conventional methods (cryotherapy, surgical excision, or no treatment) in terms of lesion regression, recurrence prevention, side effects, and posttreatment quality of life?”

I. Population: patients with clinically and histologically diagnosed OL.
II. Intervention: laser treatments (ablative, fractional, and laser‐assisted PDT).
III. Comparison: conventional therapies such as cryotherapy, surgical excision, or no treatment.
IV. Outcome: lesion regression, recurrence reduction, fewer side effects, and improved posttreatment quality of life.

### 2.3. Data Processing

Four independent reviewers (Pierluigi Marotti, Silvia Chieppa, Pietro Lauria, Daniela Di Venere, and Massimo Corsalini) assessed the quality of the included studies using specified criteria such as selection criteria, methods of outcome evaluation, and data analysis.

This enhanced “risk of bias” tool additionally includes quality standards for selection, performance, detection, reporting, and other biases.

Any differences were settled through conversation or collaboration with other researchers (Alessio Danilo Inchingolo, Andrea Palermo, Angelo Michele Inchingolo, and Gianna Dipalma). The reviewers screened the records according to the inclusion and exclusion criteria.

Doubts have been resolved by consulting the senior reviewer (Francesco Inchingolo). The selected articles were downloaded into Zotero.

## 3. Results

### 3.1. Characteristics of Included Articles

**Figure 1:**
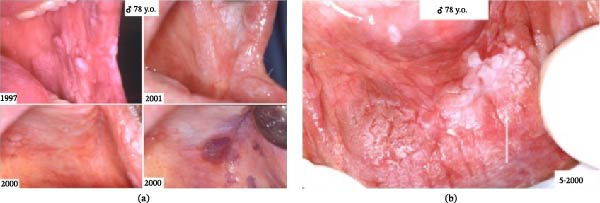
(a) Clinical presentation of oral leukoplakia. (b) Leukoplakic lesion of oral mucosa.

Figure 1 shows the flow diagram of a systematic review carried out using the PRISMA reporting criteria. The diagram describes the search strategy, inclusion, and exclusion of publications at each stage of detection.

**Figure 2:**
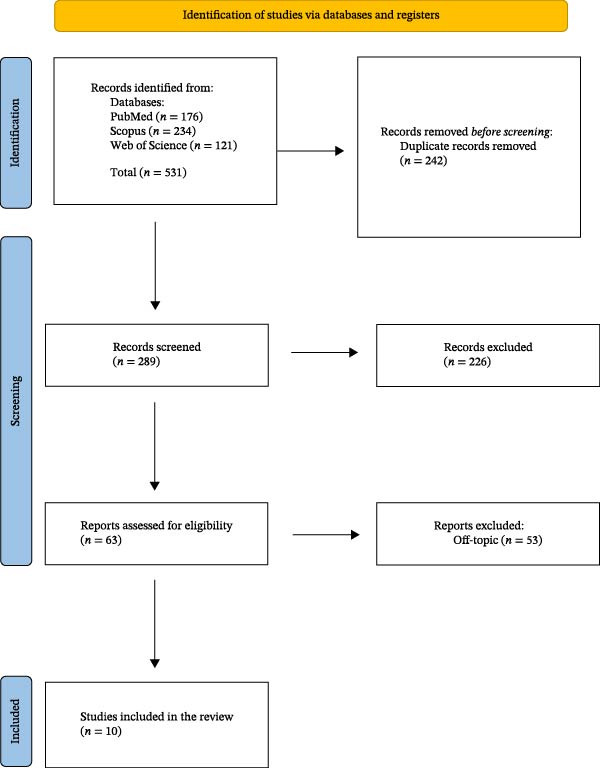
Prisma flowchart.

A total of 531 publications were identified in three databases, including PubMed (176), Web of Science (121), and Scopus (234), obtaining 289 records after the duplicates were deleted (242). The title and abstract analysis resulted in the exclusion of 226 articles because they were off‐topic. The remaining 63 records were read, deleting 53 articles that did not fill the inclusion criteria. The evaluation includes a total of 10 publications for qualitative analysis (Figure 2).

### 3.2. Quality Assessment and Risk of Bias of Included Articles

**Figure 3:**
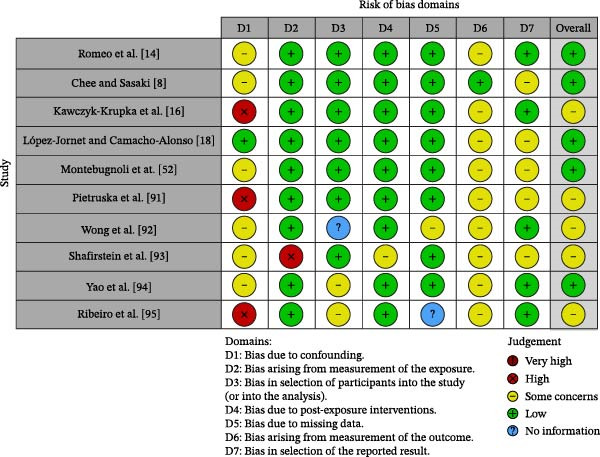
Risk of bias assessment.

The risk of bias in the included studies is reported in Figure 3.

Regarding bias due to confounding, most studies have a low risk (Table 1).

**Table 1:** Studies’ evaluation.

First author (Year)	Type of study	Aim of the study	Materials and methods	Results
Romeo et al. 2020 [14]	Randomized controlled clinical trial	To determine the optimal extension margin during CO_2_ laser ablation of oral leukoplakia and to assess its short‐term recurrence rate.	33 lesions diagnosed as oral leukoplakia in 30 patients (aged 39–79) were randomly allocated into three groups: Group A received laser ablation confined to the lesion, Group B underwent ablation with an additional 3 mm margin, and the Control group was observed without treatment. All lesions were confirmed by biopsy (showing hyperkeratosis without dysplasia), and procedures were standardized (including pretreatment elimination of risk factors and using a CO_2_ laser with defined parameters). Follow‐ups were scheduled at 1 week, 3 weeks, 3 months, and 6 months.	Complete healing was observed in 13 lesions (~59%) within the treated groups—with Group B (63.6% healing) showing a slight advantage over Group A (54.5% healing). In contrast, only 27.3% of lesions in the control group exhibited complete regression. Notably, recurrences—first evident at the 3‐week follow‐up—were more common in lesions from patients with a history of smoking.
Chee and Sasaki 2013 [8]	Randomized, single‐blinded prospective parallel‐group study	To compare the effectiveness of CO_2_ laser fiber excision with conventional cold knife excision in treating oral leukoplakia.	45 patients (aged 43–84) scheduled for oral leukoplakia excision were randomly assigned to two groups: 24 procedures using a flexible CO_2_ laser fiber and 23 using cold knife excision. Key parameters—including operative time (min/cm^2^), blood loss (g/cm^2^), frequency of bipolar cautery use (uses/cm^2^), and the number of additional margins required (assessed by frozen section)—were recorded. Additional postoperative outcomes, such as pain and time to oral intake, were also documented.	Although operative times were similar (1.64 vs. 1.70 min/cm^2^), the CO_2_ laser group had significantly lower blood loss (0.19 g/cm^2^ vs. 2.55 g/cm^2^), fewer uses of bipolar cautery (0.34 vs. 3.32 uses/cm^2^), and required fewer margins to clear the specimen (1.21 vs. 1.83) compared to the cold knife group.
Kawczyk‐Krupka et al. 2012 [16]	Comparative observational study	To compare the therapeutic efficacy of photodynamic therapy (PDT) versus cryotherapy for treating oral leukoplakia.	Patients diagnosed with oral leukoplakia were allocated into two groups. In the PDT group (48 patients), a topical photosensitizer (ALA) was applied followed by laser irradiation (using either 20% ALA with a 630 nm Diomed laser or 10% ALA with an argon‐pumped dye laser). In the cryotherapy group (37 patients), lesions were treated using a cryoprobe cooled by nitrous oxide to –89°C. Outcomes such as lesion clearance, recurrence rates, number of treatment sessions, and adverse effects were monitored over a variable follow‐up period.	In the PDT group, 35 patients (72.9%) achieved complete clearance, with 13 recurrences (27.1%) noted over 6 months. In contrast, cryotherapy produced complete responses in 33 patients (89.2%) with 9 recurrences (24.3%). Both methods were well tolerated; however, PDT was noted for its minimally invasive nature and improved esthetic outcomes.
López‐Jornet and Camacho‐Alonso 2013 [18]	Randomized clinical trial	To compare conventional cold knife surgery and CO_2_ laser excision in oral leukoplakia by evaluating postoperative pain and swelling.	48 patients with oral leukoplakia (27 males, 21 females; mean age 53.7 ± 11.7 years) were enrolled and randomized into two groups. Group 1 (*n* = 28) underwent standard cold knife excision with a 3‐mm margin and primary closure, while Group 2 (*n* = 20) received CO_2_ laser excision in continuous mode (with similar margins) and healing by second intention. Postoperative pain and swelling were recorded using a 100‐mm visual analog scale at various time points during the first week.	Patients treated with the CO_2_ laser experienced significantly lower pain and swelling during the first three postoperative days compared to the cold knife group. Both groups showed a gradual reduction of symptoms over 1 week, with no granuloma formation or malignant transformation observed during a mean follow‐up of ~28 months.
Montebugnoli et al. 2011 [52]	Prospective study	To investigate if the clinical resolution of oral leukoplakia following Nd:YAG laser therapy corresponds with restored normal epithelial function, and to identify factors contributing to recurrence risk.	13 patients with oral leukoplakia treated with Nd:YAG laser (Model 6000, Laser Sonics). Biopsies were performed before and after therapy. Ki67 expression was measured to assess cell turnover. Patients were followed monthly.	Significant reduction in Ki67 levels after treatment (from 27.4% to 17.6%, *p* < 0.05). Recurrence observed in 2 patients with persistently high Ki67 levels. Kaplan–Meier statistics showed a significant difference (chi‐square 7.3, *p* < 0.01).
Pietruska et al. 2013 [91]	Randomized clinical trial	To assess the clinical effectiveness of photodynamic therapy (PDT) in managing oral leukoplakia lesions.	23 patients (21–79 years) with 44 homogeneous, flat leukoplakia lesions confirmed histopathologically. Photodynamic therapy was performed using Photolon (chlorine‐e6 and DMSO) and a semiconductor laser (660 nm, 300 mW), delivering 90 J/cm^2^ in 10 sessions.	Significant lesion size reduction by 53.8% on average. Complete healing in 12 lesions (27.27%), partial healing in 22 lesions (50%), while 10 lesions (22.73%) remained unchanged. No adverse effects observed.
Wong et al. 2013 [92]	Randomized clinical trial	To establish the maximum tolerated dose (MTD) and identify the dose‐limiting toxicity (DLT) of ALA photodynamic therapy (PDT) for oral leukoplakia.	11 patients with histologically confirmed oral leukoplakia received ALA PDT using escalating light doses (up to 4 J/cm^2^ at 585 nm). Clinical and biological markers were evaluated.	No significant toxicity was observed up to a light dose of 4 J/cm^2^. One patient had a partial clinical response at 3 months; one experienced transient grade 3 transaminase elevation. Determination of MTD was inconclusive.
Shafirstein et al. 2011 [93]	Randomized clinical trial	To evaluate the safety and effectiveness of photodynamic therapy with 5‐aminolevulinic acid (ALA) and a pulsed dye laser for treating oral leukoplakia.	23 patients (aged 37–79) with confirmed leukoplakia received 5‐ALA followed by activation using a high‐power 585 nm pulsed dye laser. Key measures included regression rates and histological changes.	No severe adverse events; minor local effects observed. Maximum tolerated dose was 8 J/cm^2^. 41% of patients showed >75% regression, 53% had >25% regression. p53 expression increased in 73% of cases; Ki‐67 decreased in 58%.
Yao et al. 2021 [94]	Randomized controlled pilot study	To evaluate and compare the effectiveness and recurrence rates of ablative fractional laser‐assisted photodynamic therapy (AFL‐PDT) with ablative fractional laser (AFL) alone in treating oral leukoplakia.	48 patients with histologically confirmed oral leukoplakia were randomized to receive AFL‐PDT or AFL treatment. Patients were followed at 1, 3, 6, and 12 months posttreatment.	AFL‐PDT showed a 100% effective cure rate compared to 80.9% for AFL alone (*p* < 0.05). Recurrence rates were lower in the AFL‐PDT group at 6 and 12 months. No severe side effects were observed.
Ribeiro et al. 2019 [95]	Randomized clinical trial	To examine the effectiveness of low‐level laser therapy (LLLT) in reducing pain associated with the cryosurgical treatment of oral leukoplakia (OL).	18 patients with OL were divided into two groups: 10 received cryosurgery (Non‐LLLT group) and eight received cryosurgery with LLLT (LLLT group). LLLT was performed using a 660 nm GaAlAs laser. Pain was measured with a numerical rating scale.	The LLLT group reported significantly less pain than the non‐LLLT group. No recurrence was observed during the 9‐month follow‐up.

The bias resulting from measurement is a parameter with a low risk of bias. Many studies have a low risk of bias due to participant selection.

Post‐exposure bias is low in most studies. Bias due to missing data is medium in many studies. The bias resulting from the outcome measurement cannot be calculated due to heterogeneity. The selection bias of the reported results is low in more than half of the studies and medium in the others. The final results show that six studies have a low risk of bias, and four have a medium risk of bias.

#### 3.2.1. Narrative Explanation of Risk of Bias Assessment

##### 3.2.1.1. Romeo et al. [14]

The randomization process was mentioned, indicating a low risk of bias in the randomization process (D1). However, it is unclear whether blinding of surgical staff or outcome assessors was implemented, raising concerns about potential deviations from the intended interventions (D2). The objective nature of the primary outcomes (healing and recurrence) and the detailed follow‐up schedule suggest a low risk of bias in the domains of missing outcome data (D3) and outcome measurement (D4). The overall judgment is “some concerns,” primarily due to the lack of clarity regarding blinding.

##### 3.2.1.2. Chee and Sasaki [8]

Although the study was single‐blinded (toward the patient), surgeons could not be blinded to the intervention modality (laser vs. scalpel). This lack of blinding could have influenced the conduct of the intervention itself (e.g., the decision to use cautery) and introduces a high risk of bias due to deviations from the intended interventions (D2). In contrast, the primary outcomes (blood loss and operative time) are highly objective and measurable, leading to a low risk of bias in their measurement (D4) and the analysis of missing data (D3). The baseline risk of bias (D1) is low due to randomization.

##### 3.2.1.3. Kawczyk‐Krupka et al. [16]

High risk. Randomization was not adequately described (D1: some concerns). The radically different nature of the interventions (PDT vs. cryotherapy) made blinding of patients and operators impossible, introducing a high risk of performance bias (D2). Although the primary outcome (clearance) was objective (D4: low risk), the lack of a preregistered protocol raises concerns about reporting bias (D5).

##### 3.2.1.4. López‐Jornet and Camacho‐Alonso [18]

Similar to Chee et al., the lack of blinding is the main issue. Patients and assessors were not blinded to the received intervention, which has a direct and significant impact on the measurement of subjective outcomes like pain and swelling (D4: high risk). This also introduces a high risk of bias due to deviations from intended interventions (D2), as postoperative management could have been influenced by knowledge of the treatment. Randomization controls for baseline bias (D1: low risk).

##### 3.2.1.5. Montebugnoli et al. [52]

High risk. The study presents a high risk of randomization bias (D1) due to the absence of a comparable control group, making it impossible to attribute the results solely to the treatment. Domains related to treatment adherence (D2) and outcome measurement (D4) are at low risk, but the fundamental experimental design compromises overall validity.

##### 3.2.1.6. Pietruska et al. [91]

This study appears methodologically sound but lacks crucial details for a definitive assessment. Randomization was appropriate (D1: low risk). However, it was not specified whether the assessment of lesion reduction was performed by an assessor blinded to the treatment. The lack of clarity on this point raises “some concerns” for bias due to deviations from intended interventions (D2) and in outcome measurement (D4). The outcomes are well‐defined.

##### 3.2.1.7. Wong et al. [92]

This dose‐escalation study is rated as low risk. Its primary objective was to establish the maximum tolerated dose (MTD) and toxicity, which are highly objective outcomes. The nature of the study (defining a dose) minimizes the risk of bias in the selection of the reported results (D5). Randomization and careful toxicity monitoring control for bias in all other domains (D1–D4: low risk).

##### 3.2.1.8. Shafirstein et al. [93]

Similar to Pietruska et al., this study is fundamentally sound, but the lack of details on the blinding of assessors who measured lesion regression (%) introduces “some concerns” for bias due to deviations from intended interventions (D2) and in outcome measurement (D4). Randomization and the clear definition of toxicity are strengths (D1, D3: low risk).

##### 3.2.1.9. Yao et al. [94]

Although randomized (D1: low risk), it compares two very different interventions (AFL‐PDT vs. AFL alone). It is likely that patients and physicians were not blinded to the assigned treatment, which could influence expectations and postoperative care (D2: some concerns). The main concern is whether the assessor of recurrences, the primary outcome, was blinded to the treatment group. If not, the risk of bias in outcome measurement (D4) would be high.

##### 3.2.1.10. Ribeiro et al. [95]

This study has a high risk of bias due to the nature of the intervention and the outcome. Low‐level laser therapy (LLLT) is an additive, and patients knew whether they had received it or not. The primary outcome was pain, measured on a subjective scale. The combination of a subjective outcome and the lack of blinding of patients and assessors introduces a very high risk of bias in the measurement of the result (D4) and in deviations from the intended interventions (D2).

### 3.3. Risk of Bias

Table 1. Risk of bias assessment.

## 4. Discussion

The studies present a thorough and detailed analysis of different treatment approaches for OL, carefully outlining the advantages and limitations of each method [96–99]. PDT has proven to be a highly effective treatment, leading to significant clinical and histological improvements. In particular, PDT utilizing 5‐ALA has been found to greatly diminish leukoplastic lesions and restore epithelial normalcy, indicating its potential in lowering the risk of progression to oral carcinoma [100–103]. The minimal side effects associated with PDT make it an especially appealing option, particularly in preserving both the aesthetic and functional integrity of the oral mucosa [104–106].

CO_2_ laser therapy has demonstrated superiority over the conventional cold scalpel in the excision of OL, resulting in significantly less postoperative pain and swelling [93, 107–110]. This technique provides multiple advantages, such as reduced blood loss, enhanced visualization of the surgical site, and minimized tissue trauma, all contributing to a better postoperative experience for patients. However, it is essential to recognize that CO_2_ laser treatment is more costly than the traditional scalpel, necessitating a comprehensive cost–benefit assessment [111–114].

The AsGaAl laser, operating at 660 nm, has been shown to effectively reduce postoperative pain following cryosurgery, leading to significant pain relief and accelerated healing with a decreased need for analgesics [87, 115–117]. Its minimal side effects are generally well‐tolerated, suggesting that this laser treatment can substantially enhance patient quality of life during the recovery period.

CO_2_ laser ablation with margin extension has demonstrated greater efficacy than procedures without margin extension, as it promotes better epithelial normalization and lowers lesion recurrence rates. However, patients undergoing margin extension reported slightly increased postoperative pain.

Although laser and PDT therapies are generally well tolerated, some patients may still experience mild postoperative discomfort, such as localized soreness or irritation [118, 119]. Occasional reports describe transient swelling or sensitivity at the treatment site, which typically resolves without intervention [120, 121]. Other studies note that patients may experience temporary functional limitations, such as minor difficulty in eating or speaking, during the initial recovery period [122, 123].

PDT, whether performed with a pulsed dye laser or an ablative fractional laser, has shown superior effectiveness compared to cryosurgery and ablative fractional laser alone, as it decreases dysplastic changes and facilitates epithelial normalization. Additionally, this technique is associated with less pain and discomfort, making it a more tolerable option for patients. Treatment with the Nd laser has also been observed to support the regeneration of a well‐differentiated and stable epithelium, thereby reducing both recurrence rates and the risk of carcinoma progression [124–126].

### 4.1. Comparison of Treatment Methods

PDT has been shown to be an effective treatment for OL, leading to significant clinical and histological improvements. PDT with 5‐ALA has been particularly successful in reducing leukoplastic lesions and restoring normal epithelial structure, suggesting its potential role in lowering the risk of progression to oral carcinoma [127, 128]. The minimal side effects associated with PDT make it a highly favorable option, particularly for preserving the integrity of the oral mucosa [6, 86, 124, 129–131].

The CO_2_ laser has demonstrated greater effectiveness than the cold scalpel in the removal of OL, as it significantly reduces postoperative pain and swelling. This approach provides key benefits such as decreased blood loss, improved visibility of the surgical area, and minimized tissue damage, ultimately enhancing the postoperative experience for patients. However, it is important to note that CO_2_ laser treatment is more costly than the cold scalpel, necessitating a thorough cost–benefit evaluation [1, 132–137].

The AsGaAl laser at 660 nm has been found to be highly effective in minimizing postoperative pain following cryosurgery, leading to significant pain relief and faster healing with a reduced need for analgesics. The minimal side effects of this laser treatment are well tolerated, suggesting that it can significantly enhance patient quality of life during the recovery phase [138–142].

CO_2_ laser ablation with margin extension has been shown to be more effective than ablation without margin extension, resulting in better epithelial normalization and a lower recurrence rate of lesions. However, patients who underwent margin extension reported slightly higher levels of postoperative pain. These findings indicate that while margin extension can improve treatment efficacy and reduce the likelihood of carcinoma progression, the potential increase in discomfort should be carefully evaluated [143–146].

The evidence suggests that both PDT and the CO_2_ laser provide significant benefits in reducing dysplastic alterations and supporting normal epithelial regeneration, with minimal side effects. Extending margins in CO_2_ laser ablation enhances treatment effectiveness and lowers the risk of recurrence. However, larger studies with long‐term follow‐up are necessary to validate these findings and assess their long‐term impact [147–150].

Many studies had small sample sizes and short follow‐up periods. Future research involving larger patient groups and extended follow‐up durations is required to confirm these outcomes and better understand the long‐term effects on recurrence prevention and carcinoma progression. Additionally, treatment costs and the availability of specialized equipment—especially for more expensive approaches like the CO_2_ laser—must be taken into account [145, 151–155].

In conclusion, these studies provide valuable insights into the different therapeutic options for OL, emphasizing their effectiveness, benefits, and possible limitations [19, 156–159]. Further large‐scale research is essential to fully comprehend the long‐term implications and optimize treatment strategies for patients with OL [4, 129, 160–163].

## 5. Conclusion

In summary, the various treatment approaches exhibit different levels of effectiveness in managing OL [88, 163–165]. Both PDT and the CO_2_ laser present notable benefits in minimizing dysplastic changes and facilitating the regeneration of normal epithelium, all while causing minimal side effects [166]. Incorporating margin extension in CO_2_ laser ablation can enhance treatment success and lower the likelihood of recurrence; however, further long‐term studies with larger sample sizes are necessary to validate these findings and assess their prolonged effects [167–171]. Overall, the evidence indicates that PDT and the CO_2_ laser represent promising therapeutic options for OL, potentially improving patient outcomes and decreasing the risk of progression to oral carcinoma [102, 172–174].

**Nomenclature**
ALA: 5‐Aminolevulinic acid
AsGaAl: Aluminum gallium arsenide
CO_2_: Carbon dioxide
Nd: Neodymium
OL: Oral leukoplakia
OSCC: Oral squamous cell carcinoma
PDT: Photodynamic therapy
PRISMA: Preferred reporting items for systematic reviews and meta‐analyses.

## Funding

No funding was received for this manuscript.

## Conflicts of Interest

The authors declare no conflicts of interest.

## Data Availability

Data sharing is not applicable to this article, as no datasets were generated or analyzed during the current study.
